# Adult Hippocampal Neurogenesis and mRNA Expression are Altered by Perinatal Arsenic Exposure in Mice and Restored by Brief Exposure to Enrichment

**DOI:** 10.1371/journal.pone.0073720

**Published:** 2013-09-03

**Authors:** Christina R. Tyler, Andrea M. Allan

**Affiliations:** Department of Neuroscience, University of New Mexico School of Medicine, Albuquerque, New Mexico, United States of America; Radboud University, Netherlands

## Abstract

Arsenic is a common and pervasive environmental contaminant found in drinking water in varying concentrations depending on region. Exposure to arsenic induces behavioral and cognitive deficits in both human populations and in rodent models. The Environmental Protection Agency (EPA) standard for the allotment of arsenic in drinking water is in the parts-per-billion range, yet our lab has shown that 50 ppb arsenic exposure during development can have far-reaching consequences into adulthood, including deficits in learning and memory, which have been linked to altered adult neurogenesis. Given that the morphological impact of developmental arsenic exposure on the hippocampus is unknown, we sought to evaluate proliferation and differentiation of adult neural progenitor cells in the dentate gyrus after 50 ppb arsenic exposure throughout the perinatal period of development in mice (equivalent to all three trimesters in humans) using a BrdU pulse-chase assay. Proliferation of the neural progenitor population was decreased by 13% in arsenic-exposed mice, but was not significant. However, the number of differentiated cells was significantly decreased by 41% in arsenic-exposed mice compared to controls. Brief, daily exposure to environmental enrichment significantly increased proliferation and differentiation in both control and arsenic-exposed animals. Expression levels of 31% of neurogenesis-related genes including those involved in Alzheimer’s disease, apoptosis, axonogenesis, growth, Notch signaling, and transcription factors were altered after arsenic exposure and restored after enrichment. Using a concentration previously considered safe by the EPA, perinatal arsenic exposure altered hippocampal morphology and gene expression, but did not inhibit the cellular neurogenic response to enrichment. It is possible that behavioral deficits observed during adulthood in animals exposed to arsenic during development derive from the lack of differentiated neural progenitor cells necessary for hippocampal-dependent learning. This study is the first to determine the impact of arsenic exposure during development on adult hippocampal neurogenesis and related gene expression.

## Introduction

Arsenic is a naturally occurring essential trace element found in soil deposits and water. Both natural and human sources contribute to the ubiquitous presence of arsenic in the environment; as such, several millions of people are exposed to this toxic metalloid in varying concentrations depending on location. Consistent exposure to arsenic results in a myriad of problems associated with almost every organ system in the body, including problems with renal, cardiovascular, reproductive, hepatic, and neurological systems [1]. It’s well established that arsenic is a toxin in high doses and a co-carcinogen in moderate doses; the last decade of research has shown arsenic to be a neurotoxin in low doses. The current EPA standard for arsenic in water is 10 parts per billion; however, many people in rural areas and developing countries, where arsenic is not regulated, are exposed to much higher levels of arsenic in their water. Developmental and continuous arsenic exposure induces significant deficits in long-term memory in children, as measured by the Wechsler Intelligence Scale for Children (WISC) [2]. Other research has revealed considerable deficits in learning and memory after several methods of arsenic exposure in both rodent models and in humans [2]–[9]. Additionally, developmental arsenic exposure alters cerebellar morphology in the brain and disrupts cell cycle dynamics of neuroepithelial cells *in vitro* [10], [11]. Recently, we have demonstrated that developmental exposure to arsenic alters components of the hypothalamus-pituitary-adrenal (HPA) axis [12] including increased hypothalamic corticotrophin releasing hormone (CRH), altered corticosterione (CORT) secretion (both at baseline and in response to a stressor), decreased hippocampal 11β-HSD 1, and altered subcellular glucocorticoid receptor (GR) distribution in the hypothalamus. We have also reported deficits in learning tasks, including 8-way radial arm, novel object exploration, learned helplessness, and forced swim tasks, some of which are hippocampal-dependent [8], [13]. The underlying mechanism responsible for these behavioral outcomes in adulthood after developmental exposure to arsenic has not been elucidated to date.

Of the two neurogenic regions of the brain capable of continual mitosis, adult neurogenesis in the dentate gyrus of the hippocampus has been implicated as an integral component of hippocampal-dependent learning and memory [14]–[16]. Adult neurogenesis involves proliferation of a neural progenitor cell (NPC) population comprised of neural stem cells left over from development and transient amplifying cells, differentiation of daughter cells from NPC into either glia or neurons, and survival and integration of these new cells into the hippocampal circuitry. Research conducted over the last decade has shown that ablation of adult neurogenesis in the hippocampus results in substantial deficits in learning and memory and that these new neurons are required for integration of new memories [17], [18]. This budding population of cells seems particularly sensitive to environmental factors, as evidenced by decreased adult neurogenesis after exposure of several different neurotoxins, including adult or fetal exposure to alcohol [19]–[21] or nicotine [22], [23], and fetal exposure to lead [24]–[26] or methylmercury [27], [28]. This sensitivity in the hippocampus coupled with the vulnerability of the brain during development may result in an environment that is susceptible to damage even with low concentrations of toxin. However, the neurogenic environment shows plasticity to extrinsic factors like antidepressants, exercise, novelty, and environmental enrichment with toys and social interaction, all of which increase adult neurogenesis and learning [29]–[31]. Environmental enrichment also enhances cognitive performance, decreases depression and anxiety, and increases proliferation and differentiation of adult neural progenitor cells [31]–[34].

Based on our previously published behavioral data and research supporting the link between learning and memory and adult neurogenesis, we hypothesized that our environmentally relevant perinatal arsenic exposure model induces deficits in proliferation and differentiation of neural progenitor cells in the dentate gyrus of exposed animals. This deficit may be responsible for the behavioral outcomes we observe in these animals [8], [13]. We found that developmental exposure to a low concentration of arsenic (50 parts per billion, ppb) over the first three trimester equivalents significantly decreased the number of differentiated neural progenitor cells but did not affect proliferation of these cells in adult mice. Additionally, perinatal arsenic exposure altered 31% of target neurogenesis-related genes as well, including several involved in growth and differentiation in adult animals. Brief exposure to an enriched environment increased both proliferation and differentiation of neural progenitor cells in both control and arsenic-exposed animals and significantly reversed mRNA expression of aberrantly expressed neurogenesis genes. Thus, it is possible that impaired hippocampal adult neurogenesis is a candidate mechanism by which developmental arsenic exposure induces deficits in learning and memory seen in adulthood.

## Methods

*Note:* Arsenic is a known human carcinogen, and all arsenicals were handled with caution according to MSDS standards.

### Ethics Statement

All experiments were performed in accordance with protocols approved by the University of New Mexico Institutional Animal Care and Use Committee (Protocol number: 11–100679 HSC).

### Perinatal Arsenic Exposure Paradigm

**Figure 1:**
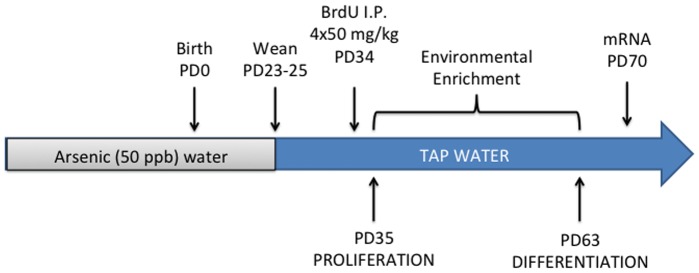
Schematic of neurogenesis study in perinatally arsenic-exposed mice. Female mice were given arsenic-treated water (50 ppb), made from sodium arsenate and ultra pure water, for seven days prior to mating. Dams drank arsenic-treated water (50 ppb) throughout pregnancy into the postnatal period. Offspring were weaned at PD23–25 and given tap water (∼5 ppb As) in standard cages. Four i.p. BrdU injections (50 mg/kg) were given over the course of 12 hours on PD34; animals were sacrificed on PD35 and proliferation of adult neurogenesis was assessed. For assessment of differentiation, a subset of animals was sacrificed four weeks later on PD63. Another cohort of animals was used for the enriched environment experiments. Brief, daily (2–4 hours) exposure to an enriched environment with a ladder, toys, housing, a running wheel, and other animals began on PD35. For proliferation assessment, animals were sacrificed five days later on PD40; for differentiation assessment, animals were sacrificed at PD63 with exposure to enrichment lasting one month. Another cohort of animals with and without exposure to enrichment was sacrificed at PD70 for mRNA assessment of neurogenesis-related genes.

The arsenic exposure paradigm was performed as previously described [13]. Briefly, C57BL/6J mice (Jackson Laboratories, Bar Harbor, ME) were bred and maintained in the University of New Mexico (UNM) Animal Research Facility on a 12-hour reverse light/dark cycle with lights off at 0800 hr. Animals had *ad libitum* access to food and water in a temperature controlled (22°C) room. Female mice were assigned to either control (arsenic-free) or arsenic exposure groups and acclimated for seven days prior to mating. Fifty parts per billion (ppb) arsenic-treated water was prepared from sodium arsenate (Sigma-Aldrich, St. Louis, MO) and ultra pure water from a Milli-Q Plus purification system (Millipore, Billerica, MA). Dams were provided *ad libitum* access to 50 ppb arsenic (equivalent to 50 µg/L) or normal tap water during breeding and pregnancy until pups were weaned at approximately PD23–25. After weaning, pups were group housed, four per cage, with *ad libitum* access to food and normal tap water (Figure 1). Male mice were used for all studies with only one animal from each litter to eliminate litter effects, *n = *7–9 mice per group. The UNM Health Sciences Center Institutional Animal Care and Use Committee approved all the procedures described in these studies. As described in our previous papers, the arsenic concentrations were 56 ppb in the arsenic-treated water and 6 ppb in standard tap water [8], [13]. Average daily water consumption is not significantly different between arsenic-exposed and control groups. We have previously assessed total arsenic concentration in whole brains of animals: perinatal arsenic exposure results in 2.24±0.2 ppb arsenic in whole brains at PD35, which is significantly greater than 1.0±0.24 ppb arsenic determined in age-matched control animals on tap water [8], [13].

### Enriched Environment Paradigm

For enrichment studies, PD35 male mice were separated into two groups: one with daily exposure to enrichment (enriched environment, EE) and one without exposure to enrichment (no enrichment, NE). Mice used for proliferation assessment were exposed to an enriched environment (EE) for 2–4 hours per day for five days after which proliferation was assessed at PD40 for both EE and NE groups. Mice used for differentiation assessment were exposed to an enriched environment for 2–4 hours per day for 28 days (approximately one month post last-BrdU injection) after which differentiation was assessed at PD63 for EE and NE groups. No enrichment control groups consisted of both perinatally arsenic-exposed animals and unexposed animals in which neither group had exposure to enrichment. The enriched environment consisted of toys (ladder, plastic house, chew toys) and a running wheel in a large housing container (48 cm×27 cm×20 cm). Toys were exchanged every other day, but the running wheel was kept throughout the exposure paradigm. The location of all the objects was changed every other day. All NE control groups (perinatal arsenic or control) and all enriched groups (perinatal arsenic or control) were maintained in standard housing (28 cm×18 cm×13 cm). Each standard housing cage contained two to three mice, while the enrichment cages contained four to six mice; the same four to six mice were exposed to enrichment together each day. All groups were given *ad libitum* access to food and tap water while in the enriched environment and in their own standard housing.

### Histological Evaluation of Neurogenesis

To analyze adult neurogenesis, 5-bromo-2′-deoxyuridine (97% Sigma-Aldrich, St. Louis, MO) BrdU was used to label neural progenitor cells (NPC) in the dentate gyrus (DG) of the hippocampus of PD34 male mice for all groups. To reduce BrdU toxicity and double labeling of neural progenitor cells, a 12-hour, 4-injection paradigm was used with a low concentration of BrdU (50 mg/kg made in 0.9% sterile NaCl) [35]. Assessment of proliferation or differentiation was performed either 12 hours (PD35) or 4 weeks (PD63) following the final BrdU injection, respectively. Brain slices were obtained according to an earlier published protocol [36]. Briefly, mice were euthanized with an i.p. injection of sodium pentobarbital followed by transcardiac perfusion with 4% PFA. Brains were dissected and post-fixed overnight in 4% PFA followed by equilibration in 30% sucrose for 48 hours. Brains were frozen in 30% sucrose and 40 µm coronal sections were obtained using a sliding microtome; tissue was stored at –20°C in 96-well plates with cryoprotectant (1∶2:2 by volume glycerol, ethylene glycol, and 0.1 M PBS, pH 7.4). Immunohistological analysis for every 1-in-6 serial, floating, 40 µm section (240 µm apart) for the rostral-caudal extent of the hippocampus was performed. Free-floating sections were washed with 0.1 M TBS (0.15 M NaCl, 3 mM KCl, 30 mM Tris-base, pH 7.4) for 15 minutes. Sections were incubated for 30 minutes in 1 N HCl at 37°C, rinsed in 0.1 M borate buffer for 30 minutes, and rinsed several times in 0.1 M TBS. After one hour incubation in TBS-T (0.1 M TBS, 0.1%Triton X, and 3% normal goat serum), sections were incubated in primary antibodies BrdU (rat, 1∶100, Abcam), Ki67 (mouse, 1∶200, Vector), NeuN (mouse, 1∶500, Millipore), doublecortin (rat, 1∶500, Cell Signaling), and DAPI (1∶1000, Sigma) in TBS-T for 72 hours at 4**°**C. Sections were washed in 0.1 M TBS and incubated for one hour in appropriate fluorescent secondary antibodies (Alexa 488; Cy5; Cy3). Sections were mounted, coverslipped with DABCO, and maintained at 4°C in the dark until analysis; a minimum of *n* = 7 was analyzed for each group.

### Stereology

StereoInvestigator software (Microbrightfield, Wiliston, VT) was used for assessment of adult neurogenesis. Both proliferation and differentiation were scored for at least 10, 1-in-6 serial sections throughout the rostral-caudal extent of the granule cell layer of the dentate gyrus for at least seven animals per group. BrdU with Ki67 positive cell counts were limited to the granule cell layer and subgranular zone next to the hilus; this was accomplished using StereoInvestigator to outline digital contours around each dentate gyrus for each section using a 10× objective. BrdU positive cells that were more than three nuclear diameters from the base of the granule cell layer or more than two nuclear diameters into the hilus were not counted, as only progenitor cells lie within this region. A two-micron guard zone was used with a 25 µm optical fractionator probe and a 40× objective for counting cells. As arsenic has been shown to induce DNA damage [37], proliferation was assessed via colocalization of BrdU positive cells (BrdU^+^) with Ki67 positive (Ki67^+^) cells using unbiased stereology. Cells expressing only BrdU or only Ki67 were not counted; although, very few cells had BrdU^+^ labeling only. One month after the final BrdU injection, differentiation of progenitors was assessed via BrdU colocalized with either doublecortin (DCX) or BrdU colocalization with NeuN positive cells. Counts for both types of differentiated cells (immature DCX^+^ and mature NeuN^+^) were pooled together to determine the total impact of arsenic exposure on the differentiation capacity of NPC. Results are presented as cell counts directly from the StereoInvestigator analysis for the total number of cells throughout the entire dentate gyrus per animal. This assessment takes into account the section sampling fraction (SSF), the area subfraction (ASF), the thickness subfraction (TSF) and the total number of cells counted (Q): N = Q/(SSF×ASF×TSF). Phenotype analysis was performed for 100 randomly selected BrdU positive cells per animal for differentiation studies. Phenotypes were counted as BrdU^+^, Brdu^+^DCX^+^, or BrdU^+^NeuN^+^.

### Confocal Microscopy

**Figure 2:**
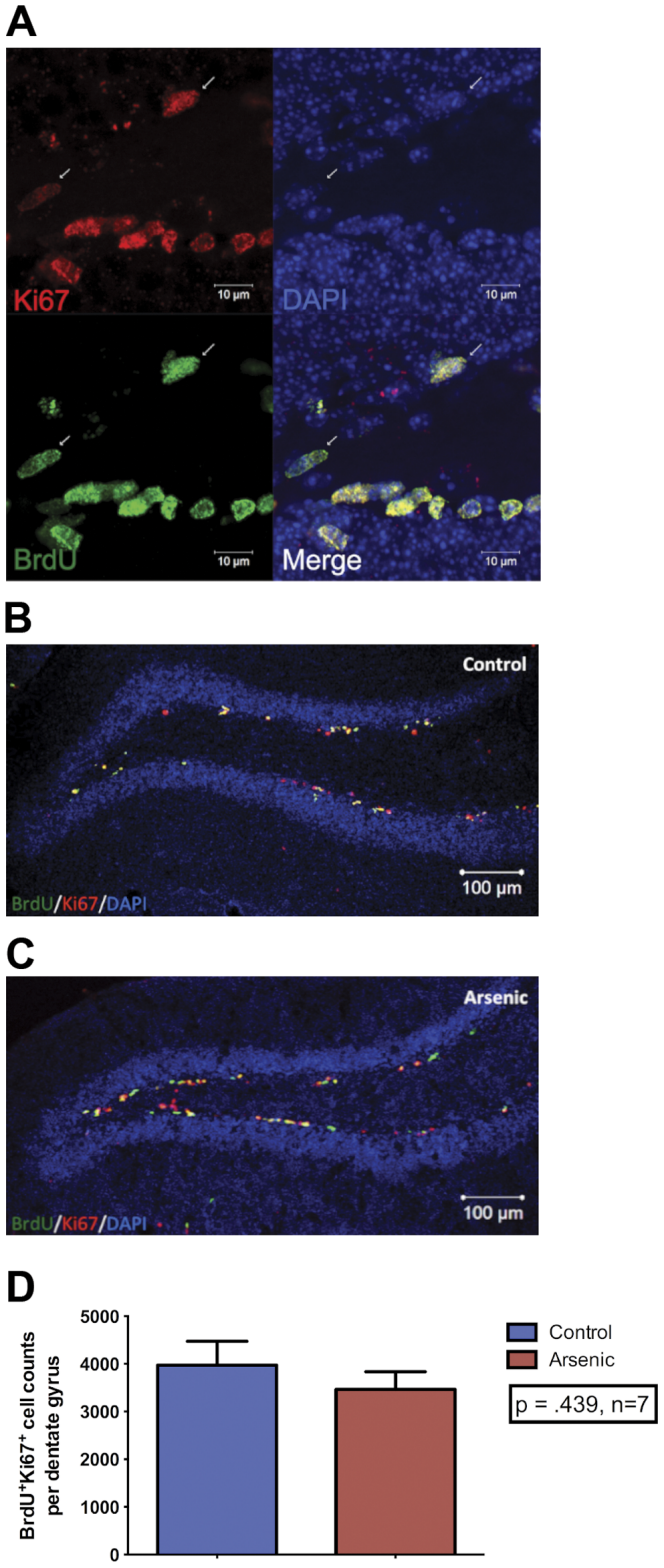
Perinatal arsenic exposure does not alter proliferation of NPC at PD35 in male mice. Representative images of neural progenitor cell (NPC) proliferation at PD35 in the dentate gyrus: (**A**) Colocalization of Ki67 (endogenous mitotic marker) and BrdU (exogenous S-phase marker) determine the proliferating pool of neural progenitor cells in (**B**) control and (**C**) arsenic-exposed animals. (**D**) There was no significant effect of treatment on the proliferation of neural progenitor cells, *n* = 7–9 mice per group (each from different litters). Results are expressed as the mean ± SEM. DAPI (blue), BrdU (green), Ki67 (red).

**Figure 3:**
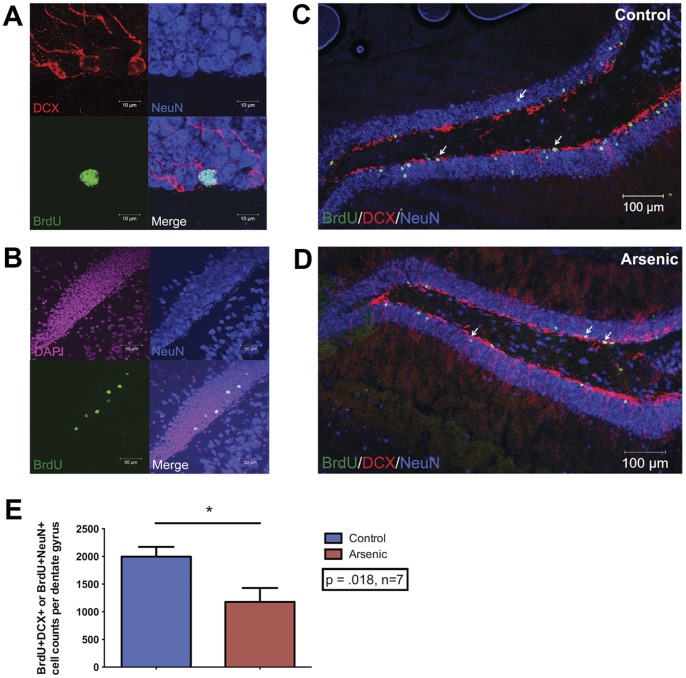
Perinatal arsenic exposure decreases NPC differentiation in PD63 male mice. (**A**) Colocalization of doublecortin (immature neuron marker DCX) or NeuN (mature neuron marker) and BrdU determine differentiated neurons derived from labeled neural progenitor cells. (**B**) Colocalization of BrdU with NeuN in the dentate gyrus reveals mature daughter cells of BrdU-labeled progenitor cells, (BrdU, green; DAPI, pink; NeuN blue). Arrows indicate colocalization of marks and cells that were counted. Representative images of neural progenitor differentiation at PD63 in the dentate gyrus of (**C**) control and (**D**) arsenic-exposed animals. (**E**) There was a significant effect of treatment on differentiation of neural progenitor cells; BrdU^+^DCX^+^ and BrdU^+^NeuN^+^ were significantly decreased after perinatal arsenic exposure, *n* = 7–9 mice per group (each from different litters). Results are expressed as the mean ± SEM. BrdU (green), DCX (red), NeuN (blue).

Images used for stereological analysis were collected using an Olympus DSU spinning disk confocal inverted IX-81 microscope endowed with argon and HeNe lasers. A Z-stack composite was obtained using a 40× objective throughout the orthogonal plane with appropriate filters for each secondary antibody for 10 slices per brain. A guard zone of 2 µm was used along with a probe of 25 µm. Cell bodies within the guard zone were not counted. Images for publication were acquired using a Zeiss LSM510 META confocal microscope endowed with a laser diode, one argon laser, and two HeNe lasers. Colocalization was visualized using a 63× oil objective (Figure 2A, 3A), while maximum intensity projections of Z-stacks were acquired with a 20× objective.

### Real-time PCR Analysis

The dentate gyrus of the hippocampus in PD70 male mice was isolated, microdissected as described by Hagihara and colleagues, snap frozen in liquid nitrogen, and stored at –80°C until use [38], [39], *n = *6 litters/group, where one mouse is used per litter to eliminate litter effects. Total RNA was extracted from homogenized frozen tissue using the QIAshredder homogenizer (Qiagen, Valencia, CA) and the RNeasy Mini Kit (Qiagen, Valencia, CA). The concentration of mRNA was assessed using a NanoDrop ND-1000 spectrophotometer (NanoDrop Technologies, Wilmington, DE); the 260/280 absorbance ratio for all RNA was ∼ 2.0. Isolated 750 ng mRNA was converted to cDNA using the RT2 First Strand Kit (SABiosciences, Frederick, MD). Real-time PCR analysis was performed using the RT2 Profiler Mouse Neurogenesis PCR Array (PAMM-404Z, SABiosciences) according to the manufacturer’s instructions on an ABI 7300 Real-Time PCR System (Applied Biosystems). No template controls and RT controls were on the microarray. Gene of interest (GOI) CT values were normalized to the average CT value of four housekeeping genes (β-actin, B2 m, GAPDH, Hsp90ab1), and subsequent ΔCT values were assessed using the comparative CT method (ΔΔCT) [40]. Results are expressed as fold change, 2^−ΔCT(GOI-T)^/2^−ΔCT(GOI-C)^, where GOI-T refers to the gene of interest in a particular treatment group and GOI-C to the same gene of interest but in the control group. Fold change was measured as the ratio of the 2^−ΔCT^ of the GOI under exposure conditions (e.g. arsenic or enrichment) to the 2^−ΔCT^ under control conditions (e.g. tap water or no enrichment) for a particular GOI. Fold regulation was calculated as −1/2^−ΔCT^ for all fold changes less than one.

### Statistical Analysis

Neurogenesis data was analyzed using one-way ANOVA (SPSS, v.19; Chicago, IL) with post-hoc analysis performed as needed and corrected for multiple comparisons with Bonferroni, *n = *7–9 mice per group. Gene expression data was analyzed using the Student’s t-test, *n = *6 mice per group with Bonferroni corrections when multiple comparisons were performed.

## Results

### Perinatal Arsenic Exposure did not Alter Proliferation of Neural Progenitor Cells

Proliferation of neural progenitor cells (NPC) was measured using colocalization of BrdU, an exogenous S-phase marker, and Ki67, an endogenous cell proliferation marker (Figure 2A). The use of two markers was necessary, as arsenic has been shown to induce DNA damage, and BrdU may label cells with DNA damage; thus, as Ki67 expression mimics that of BrdU within the proliferative zone, it was used to confirm the BrdU+ cell counts, validate the pulse chase assay, and to ensure measurement of an actively proliferating population of neural progenitors. While it is more common to label with one or the other marker, BrdU^+^ only cell counts may overestimate proliferation of NPC and Ki67^+^ only cell counts do not give an accurate birthdate for cells. However, the disadvantage of double labeling with both BrdU and Ki67 is that cell counts do not include the very small quantity of BrdU^+^ labeled cells that exit the cell cycle within 12 hours of the injections; we found this amount to be negligible compared to the total counts of BrdU^+^Ki67^+^ for proliferation. The labeling method used here provides a depiction of a population of cells that includes neural stem cells and their progeny, transient amplifying cells, which can give rise to neurons. Four injections of 50 mg/kg BrdU (*n = *7–9 mice/group) were given over a 12-hour period to PD34 male mice. Twelve hours after the last BrdU injection, the animals were sacrificed and adult neurogenesis was measured. Labeling with BrdU^+^ and Ki67^+^ resulted in an average of 3973±497 NPC in the DG of control animals and 3465±372 NPC in the arsenic-exposed animals (Figure 2B, 2C). Thus, arsenic-exposed mice have 13% less NPC than their control counterparts. Cells that were only labeled with BrdU or Ki67 or more than three nuclei away from the subgranular zone of the dentate gyrus were not counted as proliferating progenitor cells. One-way analysis of variance (ANOVA) assessing the effect of treatment (arsenic water versus tap water) on proliferation showed that average cell counts are not significantly different from one another. As such, perinatal exposure of 50 ppb arsenic (throughout all three trimester equivalents in mice) did not alter the proliferation of NPC in the DG of the hippocampus at PD35 (Figure 2D).

### Perinatal Arsenic Exposure Reduced the Number of Differentiated Neural Progenitor Cells

Differentiation of NPC was measured using colocalization BrdU with either doublecortin (DCX), a marker of neuroblasts and immature neurons, or NeuN, a marker of mature neurons (Figures 3A, 3B). Since the impact of arsenic on the differentiation capacity of NPC is not known, we assessed overall differentiation, including the generation of neuroblasts and immature neurons (DCX^+^) and mature neurons (NeuN^+^). Differentiation was measured four weeks after the last BrdU injection to allow for maturation of BrdU-labeled cells (*n = *7–9 mice/group). Figures 3A and 3B show representative images of colocalization of BrdU with DCX (BrdU^+^DCX^+^) and BrdU with NeuN (BrdU^+^NeuN^+^): both types of cell counts were used for assessment of total differentiation. Analysis of phenotype indicated that the relative amounts of each cell type were unchanged between groups: BrdU^+^ labeling (∼25%), BrdU^+^DCX^+^ (∼15%), and BrdU^+^NeuN^+^ (∼60%). From the proliferation data, a significant quantity (approximately 3500) of NPC was labeled in both control and arsenic-exposed animals. In control animals, there were 1997±178 BrdU^+^/DCX^+^ and BrdU^+^NeuN^+^ labeled cells (Figure 3C), while in the arsenic-exposed animals, there were 1178±252 BrdU^+^DCX^+^ and BrdU^+^NeuN^+^ labeled cells. This translates into a 41% decrease in the number of differentiated cells in arsenic-exposed animals as compared to their control counterparts (Figure 3D). One-way ANOVA revealed a significant effect of arsenic exposure on differentiation of NPC in the DG (F(1,13) = 7.311, p = .018). Thus, perinatal exposure of 50 ppb arsenic (throughout all three trimester equivalents in mice) reduced the number of differentiated NPC in the DG of the hippocampus at PD63 (Figure 3E).

### Exposure to an Enriched Environment Increased Proliferation of Neural Progenitor Cells in Control Animals

**Figure 4:**
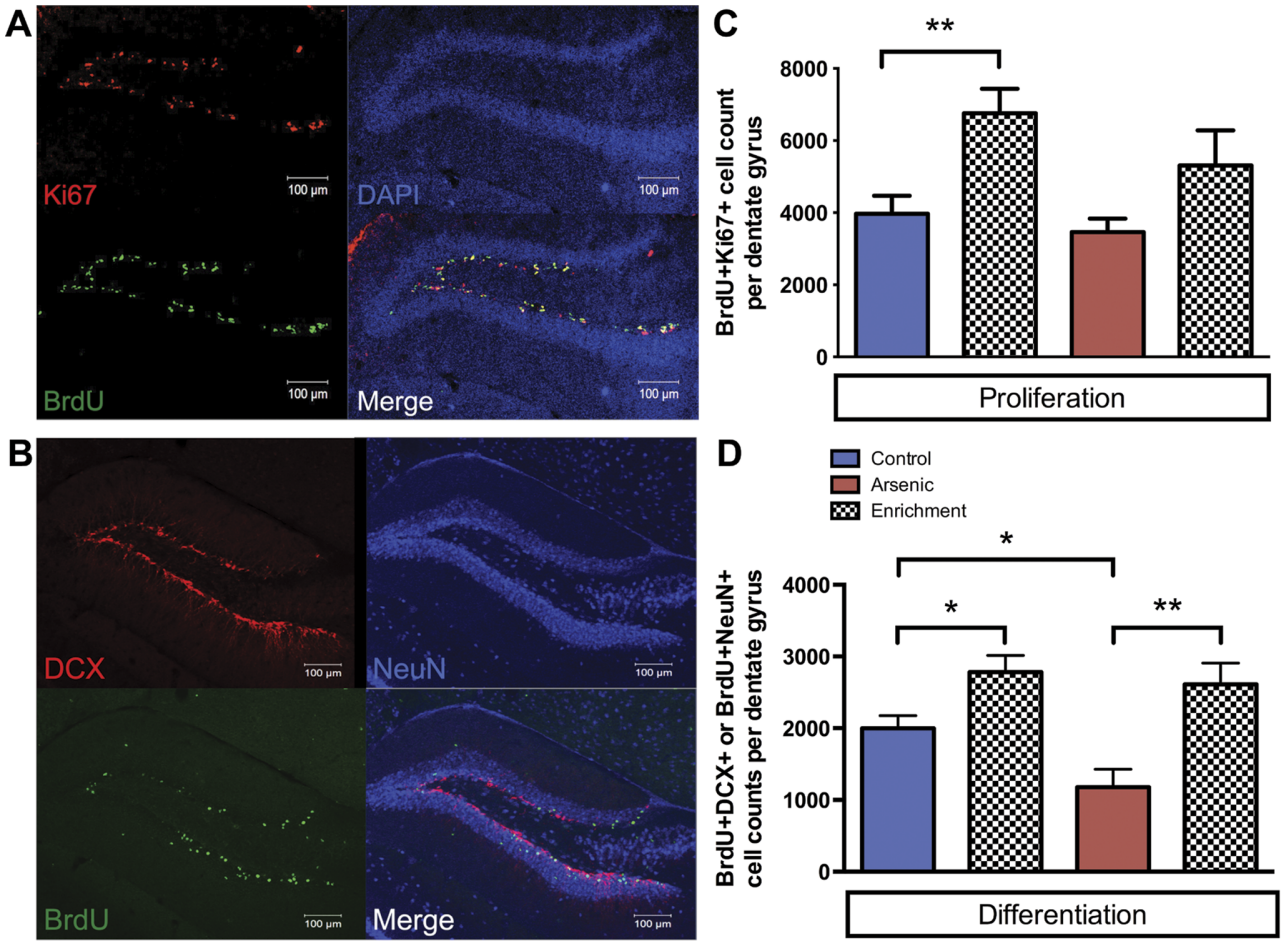
Brief, daily exposure to enrichment increases proliferation and differentiation in control and arsenic-exposed male mice. Representative images of increased (**A**) proliferation after brief exposure to enrichment for five days in arsenic-exposed animals at PD40 (BrdU (green), Ki67 (red), DAPI (blue)) and increased (**B**) differentiation after brief, daily exposure to enrichment for one month in arsenic-exposed animals at PD63 (BrdU (green), DCX (red), NeuN (blue)). (**C**) Perinatal arsenic exposure did not affect proliferation of NPC; exposure to enrichment for five days increased NPC proliferation at PD40 in both arsenic-exposed and control mice but was only significant in controls. (**D**) Perinatal arsenic exposure decreased the total number of differentiated cells (BrdU^+^DCX^+^ and BrdU^+^NeuN^+^); brief, daily exposure to enrichment for one-month increased differentiation in both arsenic-exposed and control animals at PD63, *n = *7–9 mice per group, each from different litters. (**p<.01, *p<.05).

Using the same BrdU injection paradigm, NPC were labeled in four sets of PD34 mice: control and arsenic-exposed animals that were exposed to enrichment and control and arsenic-exposed animals that were not exposed to enrichment. After five days of daily, brief (2–4 hours) exposure to enrichment after which animals were returned to their home cages, proliferation of NPC was assessed via BrdU and Ki67 colocalization in the subgranular zone of the dentate gyrus of PD40 animals (*n = *7–9 mice/group). Figure 2A shows a representative image of colocalization of BrdU with Ki67 for proliferation assessment. Assessment of proliferation resulted in 6753.64±1372.1 BrdU^+^Ki67^+^ labeled cells in control animals exposed to enrichment: this constitutes a 70% increase over control animals not exposed to enrichment. In arsenic-exposed animals exposed to enrichment, there were 5312.96±1935.95 BrdU^+^Ki67^+^ labeled cells: this constitutes a 53% increase over arsenic-exposed animals not exposed to enrichment and a 33% increase over control animals not exposed to enrichment (Figure 4A, 4C). Figure 4A shows a representative image of the DG from an arsenic-exposed animal with experience in enrichment. One-way ANOVA indicated a significant effect of enrichment on proliferation regardless of perinatal treatment (F(1,21) = 13.87, p = .001). Using a Student’s t-test with Bonferonni correction, brief exposure to enrichment slightly increased proliferation for arsenic-exposed animals but was not significant (Figure 4C, p = .061); however, exposure to enrichment did significantly increase proliferation for control mice (Figure 4C, p = .008). There is no statistically significant difference in the average cell counts between both groups exposed to enrichment. Regardless of perinatal exposure, NPC proliferation in the DG is enhanced after five days of brief exposure to enrichment, though this increase is only statistically significant in control animals.

### Exposure to an Enriched Environment Increased the Number of Differentiated Neural Progenitor Cells in All Animals

Using the same BrdU injection paradigm, NPC were labeled in four sets of PD34 mice: control and arsenic-exposed animals that were exposed to enrichment for one month and control and arsenic-exposed animals that were not exposed to enrichment (*n = *7–9 mice/group). After brief (2–4 hours), daily enrichment for 28 days, colocalization of BrdU with DCX (BrdU^+^DCX^+^) and BrdU with NeuN (BrdU^+^NeuN^+^) in the DG was assessed at PD63 in all four groups (Figure 3A, 3B). One-way ANOVA showed a significant effect of enrichment on differentiation for both controls and arsenic-exposed animals regardless of perinatal exposure (F(1,21) = 14.710, p = .001). Analysis of phenotype indicated no significant difference in the percent of cell types between the two groups exposed to enrichment (data not shown), similar to that seen in groups with no exposure to enrichment. For animals exposed to enrichment, colocalization revealed 2778±232 BrdU^+^DCX^+^ and BrdU^+^NeuN^+^ labeled cells in control mice, a 39% increase over control animals without exposure to enrichment. In arsenic-exposed mice with exposure to enrichment, colocalization revealed 2613±294 BrdU^+^DCX^+^ and BrdU^+^NeuN^+^ labeled cells, a 121% increase over arsenic-exposed animals without exposure to enrichment and a 31% increase over control animals not exposed to enrichment (Figure 4B, 4D). Figure 4B shows a representative image of the DG from an arsenic-exposed animal with one month of exposure to enrichment. Using a Student’s t-test with a Bonferroni correction, brief exposure to enrichment significantly increased differentiation for arsenic-exposed mice (Figure 4D, p = .006) and control mice (Figure 4D, p = .027). These results show that regardless of perinatal exposure, the number of differentiated NPC in the DG significantly increased after one month of brief exposure to enrichment in both control and arsenic-exposed animals.

### Perinatal Arsenic Exposure Altered mRNA Expression of Neurogenesis-related Genes

**Figure 5:**
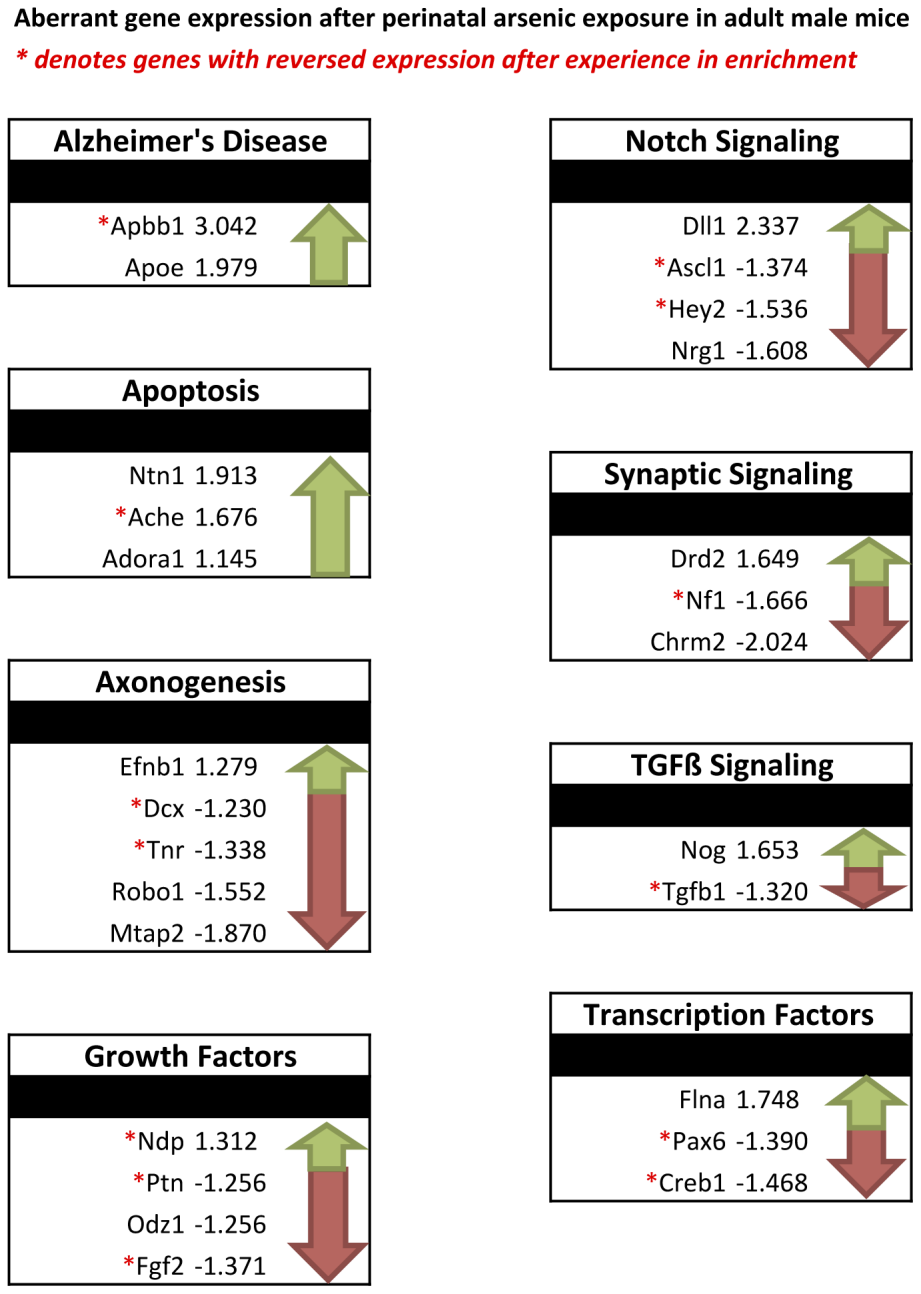
Perinatal arsenic exposure alters genes involved in several different pathways important in neurogenesis. Each box contains genes that have altered expression in the dentate gyrus derived from adult male mice exposed to 50 ppb arsenic during development. Arrows indicate direction of expression, while red asterisks indicate genes with reversed expression after exposure to environment enrichment for one month. Genes altered include those involved in Alzheimer’s disease, apoptosis, axonogenesis, growth, transcription, and Notch, synaptic, and TGFβ signaling. In each set of genes at least one aberrantly expressed gene has a reversal of expression after enrichment indicating restoration.

To analyze several genes associated with neurogenesis, we compared gene expression profiles between control and arsenic-exposed animals using a microarray on microdissected dentate gyrus tissue from PD70 animals (*n = *6 mice/group). All CT values of genes of interest (GOI) were normalized to the average CT value of four housekeeping genes (β-actin, B2 m, GAPDH, Hsp90ab1), and subsequent ΔCT values were assessed using the comparative CT method (ΔΔCT) [40]. Fold change was measured as 2^−ΔCT^(arsenic or enrichment)/2^−ΔCT^ (control or no enrichment) for each GOI. Fold regulation is expressed as the negative inverse for negative fold changes. A Student’s t-test was used to compare exposure conditions to control conditions. Table 1 shows gene expression of arsenic-exposed adult males compared to age-matched control animals. Of the 82 genes on the microarray, 13.4% were significantly upregulated (p<.05): these included Apbb1, Apoe, Ntn, Ache, and Adora1, which are involved in apoptosis and in Alzheimer’s disease. Perinatal arsenic exposure resulted in downregulation (p<.05) of 18.3% of the genes on the array. These included genes involved in axonogenesis and neurite growth (Dcx, Tnr, Robo1, Mtap2), growth factors (Ptn, Odz, Fgf2), transcription factors (Pax6 and Creb1), synaptic signaling molecules (Nf1 and Chrm2), and Notch and TGFβ signaling molecules (Ascl1, Hey2, Nrg1, and Tgfb1) as seen in Figure 5.

**Table 1:** Aberrant expression of neurogenesis-related genes in perinatal arsenic-exposed adult male mice compared to control age-matched animals.

Accession Number	Gene Title	Gene Symbol	Fold Regulation	p-value	Function
NM_009599	Acetylcholinesterase	Ache	1.676	<.01	Apoptosis; Synaptogenesis
NM_001008533	Adenosine A1 receptor	Adora1	1.145	<.05	Apoptosis; GPCR Signaling
NM_009685	Amyloid beta (A4) precursor protein-binding,family B, member 1	Apbb1	3.042	<.01	Apoptosis; Axonogenesis
NM_009696	Apolipoprotein E	Apoe	1.979	<.01	Synaptic Transmission
NM_008553	Achaete-scute complex homolog 1	Ascl1	*−*1.374	<.01	Notch Signaling
NM_203491	Cholinergic receptor, muscarinic 2, cardiac	Chrm2	*−*2.024	<.05	GPCR Signaling; Synaptic Transmission
NM_133828	CAMP responsive element binding protein 1	Creb1	*−*1.468	<.01	Synaptic Transmission; Transcription Factor
NM_010025	Doublecortin	Dcx	*−*1.230	<.05	Axonogenesis
NM_007865	Delta-like 1	Dll1	2.337	<.05	Notch Signaling; Cell Adhesion
NM_010077	Dopamine receptor D2	Drd2	1.649	<.05	Axonogenesis; GPCR Signaling; Synaptic Transmission
NM_010110	Ephrin B1	Efnb1	1.279	<.05	Cell Adhesion
NM_008006	Fibroblast growth factor 2	Fgf2	*−*1.371	<.01	Growth Factor; Synaptic Transmission
NM_010227	Filamin, alpha	Flna	1.748	<.01	Transcription Factor
NM_013904	Hairy/enhancer-of-split related withYRPW motif 2	Hey2	*−*1.536	<.05	Notch Signaling; Transcription Factor
NM_001039934	Microtubule-associated protein 2	Mtap2	*−*1.870	<.01	Axonogenesis
NM_010883	Norrie disease (pseudoglioma)	Ndp	1.312	<.01	Growth Factor; WNT Signaling
NM_010897	Neurofibromatosis 1	Nf1	*−*1.666	<.05	Synaptic Transmission
NM_008711	Noggin	Nog	1.653	<.05	Axonogenesis
NM_178591	Neuregulin 1	Nrg1	*−*1.608	<.01	Growth Factor; Notch Signaling
NM_008744	Netrin 1	Ntn1	1.913	<.05	Apoptosis
NM_011855	Odd Oz/ten-m homolog 1	Odz1	*−*1.256	<.05	Growth Factor
NM_013627	Paired box gene 6	Pax6	*−*1.390	<.05	Transcription Factor
NM_008973	Pleiotrophin	Ptn	*−*1.256	<.05	Cell Cycle; Cytokine; Growth Factor
NM_019413	Roundabout homolog 1	Robo1	*−*1.552	<.01	Cell Adhesion
NM_011577	Transforming growth factor, beta 1	Tgfb1	*−*1.320	<.05	TGFß Signaling; Cytokine
NM_022312	Tenascin R	Tnr	*−*1.338	<.01	Cell Adhesion

### Exposure to an Enriched Environment Reversed Altered mRNA Expression in Arsenic-exposed Animals

**Figure 6:**
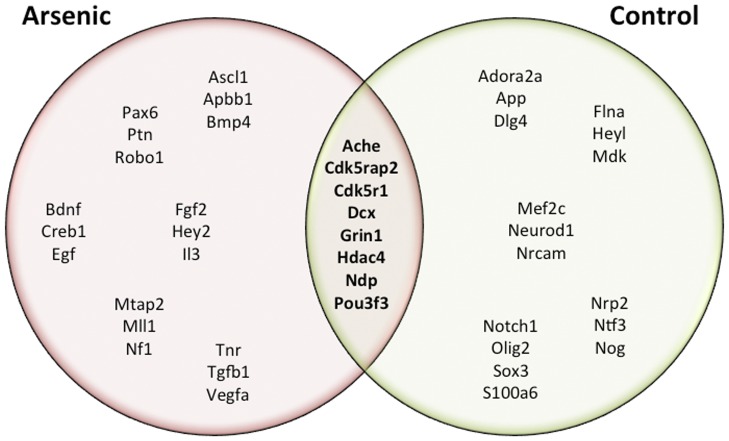
One month of brief, daily exposure to enrichment alters gene expression in both arsenic-exposed and control animals. A Venn diagram depicts gene expression alterations after brief, daily exposure to enrichment for one month in control animals (right) and in arsenic-exposed animals (left). Fold changes of displayed genes include up and down regulation, p<.05 (Tables 2, 3). Gene expression changes common to both sets of animals are displayed in the middle. These include genes involved in cell signaling, axonal growth, histone maintenance, receptors, and cell cycle dynamics.

After one month of brief, daily exposure (2–4 hours per day) to an enriched environment, mRNA expression for neurogenesis-related genes was analyzed on dentate gyrus tissue derived from adult male mice aged PD70 (*n = *6 mice/group). Genes that were significantly altered (p<.05) after this enrichment paradigm are displayed in Table 2 for control animals and Table 3 for arsenic-exposed animals. Enrichment induced several changes in both sets of animals in genes involved in growth; axonogenesis; cell cycle dynamics; transcription factors; and Notch, WNT, and TGFβ signaling. Enrichment in the arsenic-exposed mice compared to their arsenic-exposed counterparts without exposure to enrichment included significant upregulation of 23 genes (Table 3); 12 of those 23 genes were previously downregulated in arsenic-exposed animals prior to enrichment, indicating a reversal of expression due to the enrichment exposure (Table 4 column 2). These same 12 genes were upregulated in control animals with exposure to enrichment to a greater extent than in the arsenic-exposed animals (Table 4). Figure 6 shows all genes that had altered expression after exposure to environmental enrichment in arsenic-exposed animals and control animals, including those genes that were common to both groups. Complete data sets for gene expression from the array are provided in Table 5 (arsenic-exposed), Table 6 (control animals with exposure to enrichment), and Table 7 (arsenic-exposed animals with exposure to enrichment).

**Table 2:** Exposure to enrichment for one month altered gene expression in control adult male mice compared to control age-matched animals without exposure to enrichment, p-value <.05.

Accession Number	Gene Title	Gene Symbol	Fold Regulation	Function
NM_009599	Acetylcholinesterase	Ache	1.188	Apoptosis; Synaptogenesis
NM_009630	Adenosine A2a receptor	Adora2a	*−*1.527	Apoptosis; GPCR Signaling
NM_007471	Amyloid beta (A4) precursor protein	App	*−*1.203	Notch Signaling
NM_145990	CDK5 regulatory subunit associated protein 2	Cdk5rap2	*−*5.006	Cell Cycle
NM_009871	Cyclin-dependent kinase 5, regulatory subunit 1(p35)	Cdk5r1	*−*1.349	Cell Cycle
NM_007864	Discs, large homolog 4	Dlg4	*−*1.310	Synaptic Transmission
NM_010025	Doublecortin	Dcx	1.193	Axonogenesis
NM_010227	Filamin, alpha	Flna	1.426	Transcription Factors
NM_008169	Glutamate receptor, ionotropic, NMDA1	Grin1	*−*1.155	Synaptic Transmission
NM_013905	Hairy/enhancer-of-split related with YRPWmotif-like	Heyl	*−*1.494	Notch Signaling
NM_207225	Histone deacetylase 4	Hdac4	*−*1.341	Cell Cycle
NM_010784	Midkine	Mdk	1.934	Cell Cycle
NM_025282	Myocyte enhancer factor 2C	Mef2c	1.198	Transcription Factors
NM_010894	Neurogenic differentiation 1	Neurod1	2.344	Transcription Factors
NM_176930	Neuron-glia-CAM-related cell adhesion molecule	Nrcam	1.374	Synaptogenesis
NM_010939	Neuropilin 2	Nrp2	*−*1.188	Cell Adhesion
NM_008742	Neurotrophin 3	Ntf3	1.427	Synaptic Transmission
NM_008711	Noggin	Nog	*−*1.962	Axonogenesis
NM_010883	Norrie disease	Ndp	1.326	WNT Signaling
NM_008714	Notch gene homolog 1	Notch1	*−*1.422	Notch Signaling
NM_016967	Oligodendrocyte transcription factor 2	Olig2	*−*2.664	Growth Factor
NM_008900	POU domain, class 3, transcription factor 3	Pou3f3	*−*1.267	Transcription Factors
NM_009237	SRY-box containing gene 3	Sox3	*−*1.537	Transcription Factors
NM_011313	S100 calcium binding protein A6 (calcyclin)	S100a6	2.193	Axonogenesis

**Table 3:** Exposure to enrichment for one month altered gene expression in arsenic-exposed adult male mice compared to arsenic-exposed age-matched animals without exposure to enrichment, p-value <.05.

Accession Number	Gene Title	Gene Symbol	Fold Regulation	Function
NM_009599	Acetylcholinesterase	Ache	*−*1.407	Apoptosis; Synaptogenesis
NM_008553	Achaete-scute complex homolog 1	Ascl1	1.265	Notch Signaling
NM_009685	Amyloid beta (A4) precursor protein-binding, family B,member 1	Apbb1	*−*1.969	Axonogenesis
NM_007554	Bone morphogenetic protein 4	Bmp4	1.616	TGFß Signaling
NM_007540	Brain derived neurotrophic factor	Bdnf	1.251	Growth Factor
NM_133828	CAMP responsive element binding protein 1	Creb1	1.470	Transcription Factor
NM_145990	CDK5 regulatory subunit associated protein 2	Cdk5rap2	2.722	Cell Cycle
NM_009871	Cyclin-dependent kinase 5, regulatory subunit 1	Cdk5r1	1.196	Cell Cycle
NM_010025	Doublecortin	Dcx	1.278	Axonogenesis
NM_010113	Epidermal growth factor	Egf	1.670	Growth Factor
NM_008006	Fibroblast growth factor 2	Fgf2	1.657	Growth Factor
NM_008169	Glutamate receptor, ionotropic, NMDA1	Grin1	1.160	Synaptic Transmission
NM_013904	Hairy/enhancer-of-split related with YRPW motif 2	Hey2	1.426	Notch Signaling
NM_207225	Histone deacetylase 4	Hdac4	1.331	Cell Cycle
NM_010556	Interleukin 3	Il3	3.422	Cytokine
NM_001039934	Microtubule-associated protein 2	Mtap2	1.654	Axonogenesis
NM_001081049	Myeloid/lymphoid or mixed-lineage leukemia 1	Mll1	1.238	Cell Cycle
NM_010897	Neurofibromatosis 1	Nf1	1.579	Synaptic Transmission
NM_010883	Norrie disease	Ndp	*−*1.264	WNT Signaling
NM_013627	Paired box gene 6	Pax6	1.487	Transcription Factor
NM_008973	Pleiotrophin	Ptn	1.374	Cell Cycle
NM_008900	POU domain, class 3, transcription factor 3	Pou3f3	1.272	Transcription Factor
NM_019413	Roundabout homolog 1	Robo1	1.618	Cell Adhesion
NM_022312	Tenascin R	Tnr	1.402	Cell Adhesion
NM_011577	Transforming growth factor, beta 1	Tgfb1	1.357	TGFß Signaling
NM_009505	Vascular endothelial growth factor A	Vegfa	1.467	Growth Factor

**Table 4:** Reversal of altered gene expression after one month exposure to an enriched environment compared to arsenic-exposed animals without exposure to enrichment, p-value <.05.

Gene Symbol	Perinatal Arsenic Exposure*compared to controls*	Perinatal Arsenic Exposure with EE *compared to column A*	Control (no perinatal exposure) with EE *compared to column A*
Apbb1	*3.042*	*−*1.969	*−*3.697
Ache	*1.676*	*−*1.407	*−*1.287
Ndp	*1.312*	*−*1.264	*no change*
Dcx	*−1.230*	1.278	1.608
Ptn	*−1.256*	1.374	1.547
Tgfb1	*−1.320*	1.357	1.399
Tnr	*−1.338*	1.402	1.365
Fgf2	*−1.371*	1.657	1.417
Ascl1	*−1.374*	1.265	1.555
Pax6	*−1.390*	1.487	1.760
Creb1	*−1.468*	1.470	1.547
Hey2	*−1.536*	1.426	1.684
Robo1	*−1.552*	1.618	1.528
Nf1	*−1.666*	1.579	1.781
Mtap2	*−1.870*	1.654	1.942
	***Column A***		

**Table 5:** Expression of neurogenesis-related genes in arsenic-exposed adult male mice compared to control age-matched animals, entire data set including non-significant changes.

Accession Number	Gene Title	Gene Symbol	Fold Change	p-value
NM_009599	Acetylcholinesterase	Ache	1.68	0.001
NM_001008533	Adenosine A1 receptor	Adora1	1.14	0.03
NM_009630	Adenosine A2a receptor	Adora2a	1.25	0.27
NM_007439	Anaplastic lymphoma kinase	Alk	1.49	0.06
NM_009685	Amyloid beta (A4) precursor protein-binding, family B, member 1	Apbb1	3.04	0.00
NM_009696	Apolipoprotein E	Apoe	1.98	0.01
NM_007471	Amyloid beta (A4) precursor protein	App	1.13	0.22
NM_009711	Artemin	Artn	1.03	0.90
NM_008553	Achaete-scute complex homolog 1 (Drosophila)	Ascl1	0.73	0.002
NM_009741	B-cell leukemia/lymphoma 2	Bcl2	0.86	0.13
NM_007540	Brain derived neurotrophic factor	Bdnf	0.82	0.41
NM_007553	Bone morphogenetic protein 2	Bmp2	1.21	0.48
NM_007554	Bone morphogenetic protein 4	Bmp4	0.79	0.09
NM_007559	Bone morphogenetic protein 8b	Bmp8b	0.80	0.38
NM_009871	Cyclin-dependent kinase 5, regulatory subunit 1 (p35)	Cdk5r1	0.87	0.08
NM_145990	CDK5 regulatory subunit associated protein 2	Cdk5rap2	0.47	0.16
NM_203491	Cholinergic receptor, muscarinic 2, cardiac	Chrm2	0.49	0.05
NM_133828	CAMP responsive element binding protein 1	Creb1	0.68	0.01
NM_008176	Chemokine (C-X-C motif) ligand 1	Cxcl1	0.86	0.69
NM_010025	Doublecortin	Dcx	0.81	0.02
NM_007864	Discs, large homolog 4 (Drosophila)	Dlg4	1.11	0.34
NM_007865	Delta-like 1 (Drosophila)	Dll1	2.34	0.02
NM_010077	Dopamine receptor D2	Drd2	1.65	0.02
NM_007889	Dishevelled 3, dsh homolog (Drosophila)	Dvl3	1.14	0.29
NM_010110	Ephrin B1	Efnb1	1.28	0.05
NM_010113	Epidermal growth factor	Egf	0.93	0.70
NM_177821	E1A binding protein p300	Ep300	0.78	0.07
NM_001003817	V-erb-b2 erythroblastic leukemia viral oncogene homolog 2,neuro/glioblastoma derived oncogene homolog (avian)	Erbb2	1.06	0.80
NM_008006	Fibroblast growth factor 2	Fgf2	0.73	0.001
NM_010227	Filamin, alpha	Flna	1.75	0.01
NM_010275	Glial cell line derived neurotrophic factor	Gdnf	0.96	0.88
NM_008155	Glucose phosphate isomerase 1	Gpi1	1.24	0.06
NM_008169	Glutamate receptor, ionotropic, NMDA1 (zeta 1)	Grin1	1.13	0.14
NM_207225	Histone deacetylase 4	Hdac4	0.86	0.29
NM_008235	Hairy and enhancer of split 1 (Drosophila)	Hes1	1.09	0.64
NM_010423	Hairy/enhancer-of-split related with YRPW motif 1	Hey1	1.21	0.13
NM_013904	Hairy/enhancer-of-split related with YRPW motif 2	Hey2	0.65	0.03
NM_013905	Hairy/enhancer-of-split related with YRPW motif-like	Heyl	0.96	0.81
NM_010556	Interleukin 3	Il3	0.69	0.59
NM_010784	Midkine	Mdk	1.48	0.20
NM_025282	Myocyte enhancer factor 2C	Mef2c	1.32	0.07
NM_001081049	Myeloid/lymphoid or mixed-lineage leukemia 1	Mll1	0.99	0.92
NM_001039934	Microtubule-associated protein 2	Mtap2	0.53	0.01
NM_010882	Necdin	Ndn	1.84	0.14
NM_010883	Norrie disease (pseudoglioma) (human)	Ndp	1.31	0.00
NM_010894	Neurogenic differentiation 1	Neurod1	1.05	0.87
NM_010896	Neurogenin 1	Neurog1	2.19	0.06
NM_009718	Neurogenin 2	Neurog2	1.97	0.09
NM_010897	Neurofibromatosis 1	Nf1	0.60	0.04
NM_008711	Noggin	Nog	1.65	0.05
NM_008714	Notch gene homolog 1 (Drosophila)	Notch1	0.98	0.92
NM_010928	Notch gene homolog 2 (Drosophila)	Notch2	1.08	0.46
NM_013708	Nuclear receptor subfamily 2, group E, member 3	Nr2e3	1.17	0.66
NM_176930	Neuron-glia-CAM-related cell adhesion molecule	Nrcam	1.10	0.30
NM_178591	Neuregulin 1	Nrg1	0.62	0.01
NM_008737	Neuropilin 1	Nrp1	0.86	0.62
NM_010939	Neuropilin 2	Nrp2	1.02	0.79
NM_008742	Neurotrophin 3	Ntf3	1.09	0.46
NM_008744	Netrin 1	Ntn1	1.91	0.02
NM_011855	Odd Oz/ten-m homolog 1 (Drosophila)	Odz1	0.80	0.05
NM_016967	Oligodendrocyte transcription factor 2	Olig2	1.58	0.07
NM_013625	Platelet-activating factor acetylhydrolase, isoform 1b, subunit 1	Pafah1b1	0.99	0.97
NM_033620	Par-3 (partitioning defective 3) homolog (C. elegans)	Pard3	0.97	0.71
NM_008781	Paired box gene 3	Pax3	Undetermined	N/A
NM_008782	Paired box gene 5	Pax5	1.13	0.65
NM_013627	Paired box gene 6	Pax6	0.72	0.02
NM_008900	POU domain, class 3, transcription factor 3	Pou3f3	0.92	0.47
NM_011143	POU domain, class 4, transcription factor 1	Pou4f1	1.30	0.47
NM_008973	Pleiotrophin	Ptn	0.80	0.02
NM_009007	RAS-related C3 botulinum substrate 1	Rac1	1.04	0.91
NM_019413	Roundabout homolog 1 (Drosophila)	Robo1	0.64	0.01
NM_194053	Reticulon 4	Rtn4	0.97	0.76
NM_011313	S100 calcium binding protein A6 (calcyclin)	S100a6	1.43	0.25
NM_009115	S100 protein, beta polypeptide, neural	S100b	1.08	0.72
NM_009170	Sonic hedgehog	Shh	0.74	0.12
NM_178804	Slit homolog 2 (Drosophila)	Slit2	0.86	0.39
NM_011434	Superoxide dismutase 1, soluble	Sod1	1.04	0.87
NM_011443	SRY-box containing gene 2	Sox2	1.59	0.07
NM_009237	SRY-box containing gene 3	Sox3	1.00	0.99
NM_011486	Signal transducer and activator of transcription 3	Stat3	0.90	0.21
NM_011577	Transforming growth factor, beta 1	Tgfb1	0.76	0.03
NM_009377	Tyrosine hydroxylase	Th	0.90	0.69
NM_022312	Tenascin R	Tnr	0.75	0.001
NM_009505	Vascular endothelial growth factor A	Vegfa	0.78	0.08

**Table 6:** Expression of neurogenesis-related genes in control adult male mice after brief, daily exposure to enrichment for one month compared to control age-matched animals without exposure to enrichment.

Accession Number	Gene Title	Gene Symbol	Fold Change	p-value
NM_009599	Acetylcholinesterase	Ache	1.19	0.03
NM_001008533	Adenosine A1 receptor	Adora1	0.94	0.26
NM_009630	Adenosine A2a receptor	Adora2a	0.65	0.01
NM_007439	Anaplastic lymphoma kinase	Alk	0.68	0.07
NM_009685	Amyloid beta (A4) precursor protein-binding, family B, member 1	Apbb1	0.75	0.49
NM_009696	Apolipoprotein E	Apoe	0.85	0.54
NM_007471	Amyloid beta (A4) precursor protein	App	0.83	0.05
NM_009711	Artemin	Artn	1.17	0.45
NM_008553	Achaete-scute complex homolog 1 (Drosophila)	Ascl1	1.03	0.64
NM_009741	B-cell leukemia/lymphoma 2	Bcl2	1.05	0.54
NM_007540	Brain derived neurotrophic factor	Bdnf	1.66	0.10
NM_007553	Bone morphogenetic protein 2	Bmp2	0.96	0.86
NM_007554	Bone morphogenetic protein 4	Bmp4	1.39	0.09
NM_007559	Bone morphogenetic protein 8b	Bmp8b	0.90	0.51
NM_009871	Cyclin-dependent kinase 5, regulatory subunit 1 (p35)	Cdk5r1	0.74	0.0003
NM_145990	CDK5 regulatory subunit associated protein 2	Cdk5rap2	0.20	0.03
NM_203491	Cholinergic receptor, muscarinic 2, cardiac	Chrm2	0.70	0.19
NM_133828	CAMP responsive element binding protein 1	Creb1	0.96	0.57
NM_008176	Chemokine (C-X-C motif) ligand 1	Cxcl1	0.63	0.12
NM_010025	Doublecortin	Dcx	1.19	0.04
NM_007864	Discs, large homolog 4 (Drosophila)	Dlg4	0.76	0.03
NM_007865	Delta-like 1 (Drosophila)	Dll1	0.65	0.30
NM_010077	Dopamine receptor D2	Drd2	0.76	0.28
NM_007889	Dishevelled 3, dsh homolog (Drosophila)	Dvl3	1.01	0.92
NM_010110	Ephrin B1	Efnb1	0.84	0.08
NM_010113	Epidermal growth factor	Egf	1.30	0.13
NM_177821	E1A binding protein p300	Ep300	1.01	0.91
NM_001003817	V-erb-b2 erythroblastic leukemia viral oncogene homolog 2,neuro/glioblastoma derived oncogene homolog (avian)	Erbb2	0.92	0.44
NM_008006	Fibroblast growth factor 2	Fgf2	0.94	0.62
NM_010227	Filamin, alpha	Flna	1.43	0.01
NM_010275	Glial cell line derived neurotrophic factor	Gdnf	0.91	0.72
NM_008155	Glucose phosphate isomerase 1	Gpi1	1.02	0.71
NM_008169	Glutamate receptor, ionotropic, NMDA1 (zeta 1)	Grin1	0.87	0.05
NM_207225	Histone deacetylase 4	Hdac4	0.75	0.04
NM_008235	Hairy and enhancer of split 1 (Drosophila)	Hes1	1.15	0.15
NM_010423	Hairy/enhancer-of-split related with YRPW motif 1	Hey1	0.96	0.64
NM_013904	Hairy/enhancer-of-split related with YRPW motif 2	Hey2	1.00	1.00
NM_013905	Hairy/enhancer-of-split related with YRPW motif-like	Heyl	0.67	0.02
NM_010556	Interleukin 3	Il3	0.62	0.54
NM_010784	Midkine	Mdk	1.93	0.04
NM_025282	Myocyte enhancer factor 2C	Mef2c	1.20	0.04
NM_001081049	Myeloid/lymphoid or mixed-lineage leukemia 1	Mll1	1.15	0.07
NM_001039934	Microtubule-associated protein 2	Mtap2	0.95	0.70
NM_010882	Necdin	Ndn	0.98	0.94
NM_010883	Norrie disease (pseudoglioma) (human)	Ndp	1.33	0.003
NM_010894	Neurogenic differentiation 1	Neurod1	2.34	0.03
NM_010896	Neurogenin 1	Neurog1	1.09	0.88
NM_009718	Neurogenin 2	Neurog2	0.55	0.19
NM_010897	Neurofibromatosis 1	Nf1	0.97	0.88
NM_008711	Noggin	Nog	0.51	0.04
NM_008714	Notch gene homolog 1 (Drosophila)	Notch1	0.70	0.01
NM_010928	Notch gene homolog 2 (Drosophila)	Notch2	1.09	0.37
NM_013708	Nuclear receptor subfamily 2, group E, member 3	Nr2e3	0.50	0.09
NM_176930	Neuron-glia-CAM-related cell adhesion molecule	Nrcam	1.37	0.01
NM_178591	Neuregulin 1	Nrg1	1.05	0.75
NM_008737	Neuropilin 1	Nrp1	1.46	0.10
NM_010939	Neuropilin 2	Nrp2	0.84	0.06
NM_008742	Neurotrophin 3	Ntf3	1.43	0.004
NM_008744	Netrin 1	Ntn1	1.43	0.20
NM_011855	Odd Oz/ten-m homolog 1 (Drosophila)	Odz1	0.87	0.18
NM_016967	Oligodendrocyte transcription factor 2	Olig2	0.38	0.02
NM_013625	Platelet-activating factor acetylhydrolase, isoform 1b, subunit 1	Pafah1b1	1.60	0.13
NM_033620	Par-3 (partitioning defective 3) homolog (C. elegans)	Pard3	1.15	0.12
NM_008781	Paired box gene 3	Pax3	Undetermined	N/A
NM_008782	Paired box gene 5	Pax5	0.90	0.69
NM_013627	Paired box gene 6	Pax6	1.15	0.25
NM_008900	POU domain, class 3, transcription factor 3	Pou3f3	0.79	0.002
NM_011143	POU domain, class 4, transcription factor 1	Pou4f1	0.74	0.47
NM_008973	Pleiotrophin	Ptn	1.12	0.26
NM_009007	RAS-related C3 botulinum substrate 1	Rac1	1.65	0.08
NM_019413	Roundabout homolog 1 (Drosophila)	Robo1	0.90	0.29
NM_194053	Reticulon 4	Rtn4	0.94	0.37
NM_011313	S100 calcium binding protein A6 (calcyclin)	S100a6	2.19	0.02
NM_009115	S100 protein, beta polypeptide, neural	S100b	1.45	0.11
NM_009170	Sonic hedgehog	Shh	0.81	0.21
NM_178804	Slit homolog 2 (Drosophila)	Slit2	0.72	0.07
NM_011434	Superoxide dismutase 1, soluble	Sod1	1.37	0.21
NM_011443	SRY-box containing gene 2	Sox2	0.81	0.45
NM_009237	SRY-box containing gene 3	Sox3	0.65	0.01
NM_011486	Signal transducer and activator of transcription 3	Stat3	0.93	0.26
NM_011577	Transforming growth factor, beta 1	Tgfb1	0.97	0.79
NM_009377	Tyrosine hydroxylase	Th	0.59	0.13
NM_022312	Tenascin R	Tnr	0.93	0.32
NM_009505	Vascular endothelial growth factor A	Vegfa	0.82	0.02

entire data set including non-significant changes.

**Table 7:** Expression of neurogenesis-related genes in arsenic-exposed adult male mice after brief, daily exposure to enrichment for one month compared to arsenic-exposed age-matched animals without exposure to enrichment.

Accession Number	Gene Title	Gene Symbol	Fold Change	p-value
NM_009599	Acetylcholinesterase	Ache	0.71	0.001
NM_001008533	Adenosine A1 receptor	Adora1	1.04	0.49
NM_009630	Adenosine A2a receptor	Adora2a	1.11	0.62
NM_007439	Anaplastic lymphoma kinase	Alk	0.95	0.77
NM_009685	Amyloid beta (A4) precursor protein-binding, family B, member 1	Apbb1	0.51	0.01
NM_009696	Apolipoprotein E	Apoe	0.76	0.18
NM_007471	Amyloid beta (A4) precursor protein	App	0.98	0.84
NM_009711	Artemin	Artn	1.10	0.57
NM_008553	Achaete-scute complex homolog 1 (Drosophila)	Ascl1	1.27	0.03
NM_009741	B-cell leukemia/lymphoma 2	Bcl2	1.15	0.23
NM_007540	Brain derived neurotrophic factor	Bdnf	1.25	0.05
NM_007553	Bone morphogenetic protein 2	Bmp2	1.08	0.69
NM_007554	Bone morphogenetic protein 4	Bmp4	1.62	0.001
NM_007559	Bone morphogenetic protein 8b	Bmp8b	2.04	0.10
NM_009871	Cyclin-dependent kinase 5, regulatory subunit 1 (p35)	Cdk5r1	1.20	0.02
NM_145990	CDK5 regulatory subunit associated protein 2	Cdk5rap2	2.72	0.05
NM_203491	Cholinergic receptor, muscarinic 2, cardiac	Chrm2	1.65	0.15
NM_133828	CAMP responsive element binding protein 1	Creb1	1.47	0.01
NM_008176	Chemokine (C-X-C motif) ligand 1	Cxcl1	0.66	0.44
NM_010025	Doublecortin	Dcx	1.28	0.03
NM_007864	Discs, large homolog 4 (Drosophila)	Dlg4	1.19	0.21
NM_007865	Delta-like 1 (Drosophila)	Dll1	0.69	0.21
NM_010077	Dopamine receptor D2	Drd2	0.77	0.25
NM_007889	Dishevelled 3, dsh homolog (Drosophila)	Dvl3	1.01	0.96
NM_010110	Ephrin B1	Efnb1	0.89	0.37
NM_010113	Epidermal growth factor	Egf	1.67	0.03
NM_177821	E1A binding protein p300	Ep300	1.28	0.09
NM_001003817	V-erb-b2 erythroblastic leukemia viral oncogene homolog 2,neuro/glioblastoma derived oncogene homolog (avian)	Erbb2	1.27	0.19
NM_008006	Fibroblast growth factor 2	Fgf2	1.66	0.00
NM_010227	Filamin, alpha	Flna	0.86	0.34
NM_010275	Glial cell line derived neurotrophic factor	Gdnf	1.38	0.33
NM_008155	Glucose phosphate isomerase 1	Gpi1	0.90	0.19
NM_008169	Glutamate receptor, ionotropic, NMDA1 (zeta 1)	Grin1	1.16	0.04
NM_207225	Histone deacetylase 4	Hdac4	1.33	0.05
NM_008235	Hairy and enhancer of split 1 (Drosophila)	Hes1	0.90	0.51
NM_010423	Hairy/enhancer-of-split related with YRPW motif 1	Hey1	0.96	0.77
NM_013904	Hairy/enhancer-of-split related with YRPW motif 2	Hey2	1.43	0.05
NM_013905	Hairy/enhancer-of-split related with YRPW motif-like	Heyl	1.09	0.56
NM_010556	Interleukin 3	Il3	3.42	0.04
NM_010784	Midkine	Mdk	0.70	0.20
NM_025282	Myocyte enhancer factor 2C	Mef2c	0.80	0.25
NM_001081049	Myeloid/lymphoid or mixed-lineage leukemia 1	Mll1	1.24	0.02
NM_001039934	Microtubule-associated protein 2	Mtap2	1.65	0.01
NM_010882	Necdin	Ndn	0.71	0.18
NM_010883	Norrie disease (pseudoglioma) (human)	Ndp	0.79	0.03
NM_010894	Neurogenic differentiation 1	Neurod1	0.85	0.44
NM_010896	Neurogenin 1	Neurog1	0.88	0.75
NM_009718	Neurogenin 2	Neurog2	0.77	0.40
NM_010897	Neurofibromatosis 1	Nf1	1.58	0.01
NM_008711	Noggin	Nog	0.82	0.40
NM_008714	Notch gene homolog 1 (Drosophila)	Notch1	1.24	0.21
NM_010928	Notch gene homolog 2 (Drosophila)	Notch2	1.11	0.45
NM_013708	Nuclear receptor subfamily 2, group E, member 3	Nr2e3	1.40	0.48
NM_176930	Neuron-glia-CAM-related cell adhesion molecule	Nrcam	0.91	0.08
NM_178591	Neuregulin 1	Nrg1	1.24	0.16
NM_008737	Neuropilin 1	Nrp1	1.18	0.35
NM_010939	Neuropilin 2	Nrp2	1.22	0.22
NM_008742	Neurotrophin 3	Ntf3	0.88	0.24
NM_008744	Netrin 1	Ntn1	0.80	0.22
NM_011855	Odd Oz/ten-m homolog 1 (Drosophila)	Odz1	1.26	0.12
NM_016967	Oligodendrocyte transcription factor 2	Olig2	0.89	0.62
NM_013625	Platelet-activating factor acetylhydrolase, isoform 1b, subunit 1	Pafah1b1	0.85	0.48
NM_033620	Par-3 (partitioning defective 3) homolog (C. elegans)	Pard3	1.29	0.09
NM_008781	Paired box gene 3	Pax3	Undetermined	N/A
NM_008782	Paired box gene 5	Pax5	0.93	0.76
NM_013627	Paired box gene 6	Pax6	1.49	0.02
NM_008900	POU domain, class 3, transcription factor 3	Pou3f3	1.27	0.03
NM_011143	POU domain, class 4, transcription factor 1	Pou4f1	1.33	0.48
NM_008973	Pleiotrophin	Ptn	1.37	0.0003
NM_009007	RAS-related C3 botulinum substrate 1	Rac1	0.87	0.54
NM_019413	Roundabout homolog 1 (Drosophila)	Robo1	1.62	0.01
NM_194053	Reticulon 4	Rtn4	1.13	0.14
NM_011313	S100 calcium binding protein A6 (calcyclin)	S100a6	0.69	0.12
NM_009115	S100 protein, beta polypeptide, neural	S100b	0.97	0.88
NM_009170	Sonic hedgehog	Shh	0.91	0.21
NM_178804	Slit homolog 2 (Drosophila)	Slit2	1.04	0.72
NM_011434	Superoxide dismutase 1, soluble	Sod1	0.87	0.38
NM_011443	SRY-box containing gene 2	Sox2	0.89	0.63
NM_009237	SRY-box containing gene 3	Sox3	1.37	0.14
NM_011486	Signal transducer and activator of transcription 3	Stat3	1.18	0.10
NM_011577	Transforming growth factor, beta 1	Tgfb1	1.36	0.0003
NM_009377	Tyrosine hydroxylase	Th	1.61	0.27
NM_022312	Tenascin R	Tnr	1.40	0.001
NM_009505	Vascular endothelial growth factor A	Vegfa	1.47	0.01

entire data set including non-significant changes.

## Discussion

It is widely recognized that high levels of arsenic produce deleterious effects on the brain. The results presented here demonstrate that our developmental arsenic exposure model, which represents a low and environmentally relevant concentration previously considered safe, significantly impacts adult neurogenesis, specifically the number of differentiated NPC in the SGZ. Given that our labeling method is more likely to underestimate the total degree of neurogenesis [35] (as it does not include BrdU^+^ only cells that have exited the cell cycle after 12 hours for proliferation assessment), the fact that we were able to discern a difference of 13% for proliferation (though not significant) and 41% for differentiation after perinatal arsenic exposure in the adult animal is substantive. Our subsequent studies will further elucidate arsenic’s impact on the immature cells (DCX^+^) and mature cells (NeuN^+^) separately and on NPC survival. Because adult neurogenesis has been shown to be integral for certain forms of learning and memory, the hippocampal damage measured in this study may be responsible for the cognitive deficits previously observed in this arsenic exposure model.

Other heavy metals similar to arsenic have been implicated in reduced adult neurogenesis and deficits in cognition. A single injection of 5 µg methylmercury per gram body weight at PD7, resulting in 500 ppb concentration in the brain, was enough to reduce adult neurogenesis and hippocampal size and induce hippocampal-dependent learning and memory deficits specifically in a spatial learning task [27]. Using tritiated thymidine incorporation at PD7, the authors were also able to show inhibited hippocampal DNA synthesis, degradation of cyclin E, and reduced cyclin D1 and D3 during postnatal development in which neurogenesis is highly active in the hippocampus [27]. Mercury is a potent neurotoxin and elevated exposures may lead to mental retardation; yet, this brief exposure resulted in significant morphological changes and learning deficits similar to the results we report here using an arsenic concentration an order of magnitude lower than that used in the methylmercury study. Similarly, postnatal exposure to low levels of lead (0.2% lead acetate resulting in 6 ng Pb/g brain by PD80) from PD1 to PD30 results in increased anxiety behavior, deficits in contextual fear conditioning, reduced adult neurogenesis (specifically differentiation), and preferential astroglial lineage in PD80 Wistar rats [24]. Another study using the same low level of lead acetate exposure from GD16 to PD21, resulting in 35–40 ppm Pb in blood, showed altered differentiation of adult neurogenesis at PD110 but no learning deficits in the Morris water maze [26]. Chronic exposure during development using an environmentally relevant dose of lead (1500 ppm lead acetate resulting in 258 ppm Pb in blood) results in significantly decreased proliferation and survival of granule cells and reduced mossy fiber input into CA3 region of the hippocampus from the dentate, possibly accounting for the deficits in synaptic plasticity and learning seen in lead-exposed animals [25]. Our results reveal that like mercury and lead, developmental arsenic exposure can induce potent morphological damage in the adult hippocampus even at a low concentration, and these deficits may be linked to altered behavioral changes seen in our model as we have previously reported [8], [13].

*In vitro* studies investigating the effects of developmental arsenic exposure in the parts per billion range (up to 4 µM) have shown altered cell cycling including reduced viability, minimal apoptosis, and an increase in caspase 3/7 resulting in inhibition of cell cycle progression in primary embryonic rat midbrain neuroepithelial cells [11]. Other *in vitro* work with P19 mouse embryonic stem cells (ESC) has demonstrated that low concentrations of arsenic in the ppb range (0.1 up to 1.0 µM sodium arsenite) suppress the differentiation, but not proliferation, of ESC into neurons indicated by reduced Tuj1, neurogenin 1, neurogenin 2, and NeuroD expression in arsenic-treated cells [41]. The results from these studies concur with our *in vivo* developmental model of arsenic exposure and the work presented here, suggesting that arsenic impacts the number of differentiated cells among several cell types both *in vivo* and *in vitro.*

Exposure to higher concentrations of arsenic (ppm range up to 68 mg/L) during adulthood results in changes in synaptic plasticity components, including the expression of the NMDA receptor subunit NR2A, PSD-95, and pCaMKIIα in the hippocampus, all of which are important for learning and memory [42]. In accordance with our own previous results [43], increased SynGAP, a negative regulator of Ras/MAPK activity, along with decreased pERK1/2 activity is also seen after chronic low dose arsenic exposure in the adult. Two months of 4.0 mg/L As_2_O_3_ (ppm range) exposure beginning at four months of age decreases both proliferation and differentiation of newly labeled cells in the dentate gyrus in mice [44]. This deficit was ameliorated after two months of drinking distilled water, suggesting that the effects of arsenic are transient. Our model indicates that significant changes in differentiation can be measured several weeks after developmental 50 ppb arsenic exposure, suggesting that the effect is not transient. It is possible that because arsenic is present during development of the brain, arsenic’s effects are much longer lasting than those seen during adulthood exposure due to arsenic-induced molecular changes. For example, low concentrations of arsenic during development impact the epigenetic status of histones and DNA, which play an important role in gene expression regulation; these epigenetic changes are concurrent with learning deficits seen in certain behavior tasks [45], [46]. Thus, we hypothesize that behavioral deficits seen during adulthood of mice developmentally exposed to arsenic are due, in part, to lack of differentiated cells necessary for hippocampal-dependent learning.

To support the differences in adult neurogenesis we observed in arsenic-exposed animals, we sought to elucidate if there was transcriptional dysregulation of genes involved in adult neurogenesis in the dentate gyrus. In accordance with our morphological findings, arsenic exposure during the perinatal period resulted in downregulation of 15 target genes (18%) and upregulation of 11 target genes (13%) in adult animals.

Upregulation of genes included those involved with apoptosis (Ntn1, Adora1, and Ache) and Alzheimer’s disease (ApoE, Apbb1). In particular, acetylcholinesterase (Ache) is an important regulator of apoptosis; increased expression, while not initiating apoptosis, has been correlated with apoptotic cells [47]. Adult arsenic exposure (20 mg/kg body weight for 28 days in rats) results in decreased activity of acetylcholinesterase in the hippocampus, but also decreased binding of (3)H-QNB, a label of muscarinic-cholinergic receptors [48]. While the increase in Ache mRNA is not indicative of greater activity of the enzyme, it could be overcompensating for a disruption in cholinergic signaling as suggested by the significantly reduced Chrm2 mRNA, a muscarinic cholinergic receptor (Table 1). The hippocampus receives several cholinergic inputs that may contribute to the formation of new neurons; additionally, cholinergic transmission is important for learning and memory and altered acetylcholine levels have been associated Alzheimer’s-related cognitive deficits [49]. Thus, aberrant cholinergic transmission induced by arsenic could play a part in reduced adult neurogenesis seen in our studies, along with upregulation of the genes Apbb1 and ApoE, both of which are associated with Alzheimer’s disease and have been shown to impair adult neurogenesis [50].

Additionally, while adult exposure to low concentrations of arsenic does not induce apoptosis in the hippocampus [44], developmental exposure to arsenic increases apoptosis in conjunction with decreased Nissl body staining in neuronal bodies in the hippocampus [51]. In addition to increased apoptosis after developmental arsenic exposure, a study demonstrated significant downregulation of nerve growth factor (NGF) and GAP-43 mRNA [51] in response to arsenic, similar to the results from our arrays showing significant downregulation of mRNA from Ptn, Odz1, and Fgf2. These growth factors are also involved in axonogenesis and neurite outgrowth, which arsenic exposure can impair [52]. Similarly, several important genes responsible for proper maturation and axonal growth including doublecortin, tenascin R, Mtap2, and Robo1 are all significantly downregulated in arsenic-exposed animals (Table 1). This further supports the notion that arsenic is impacting the ability of NPC to develop into mature neurons, possibly leading to the decrease in differentiated neurons in the dentate gyrus. It should be noted that the number of surviving neural progenitors does affect the number of differentiated cells as well; however, NPC survival was not assessed after four weeks. Thus, it is unclear whether this lack of differentiated cells is due to deficits in the surviving NPC population or issues with differentiation program; however, the gene expression data do suggest that arsenic exposure is impacting neurogenesis programs in the dentate gyrus.

Several pathways, including Notch, TGFβ, and WNT, all had factors that were aberrantly expressed in the arsenic-exposed animals (Table 1). Interestingly, arsenic induced an upregulation of Dll1, a ligand that promotes Notch signaling, which enhances proliferation of progenitor cells. However, increased Dll1 also drives the downregulation of Ascl1, which was also downregulated in arsenic-exposed animals. Ascl1, or Mash1, is a basic helix-loop-helix (bHLH) transcription factor that increases expression of neurogenins to promote neuronal differentiation programs [53]. Thus, the Notch signaling program may be enhanced at the expense of the differentiation of progenitors as reflected in the deficits in differentiation of NPCs in arsenic-exposed animals [54]. Arsenic exposure resulted in downregulation of other factors important for differentiation including Odz and Nrg1 (also involved in Notch signaling) and a trend toward downregulation of neurogenins 1 and 2, though not significant. Thus, all of these changes in genes important for proper differentiation could be contributing to the lack of differentiated cells we observed after developmental arsenic exposure.

It’s well established that exposure to an enriched environment can lead to increased adult neurogenesis, thereby enhancing certain forms of learning and memory [29], [32]. Yet, our previous publications have demonstrated that developmental toxins, such as alcohol, reduce the ability of the neurogenic environment in the hippocampus to respond to a continuous enrichment paradigm [55]. Other studies reveal that brief exposure to enrichment is adequate to improve behavior and adult neurogenesis [56]–[58]. To reduce the likelihood that animals habituate to the enriched housing environment, we decided to use a “playground” enrichment paradigm in which animals spend two to four hours per day in the enrichment cages (with toys, ladders, running wheels, housing, and other animals) and are subsequently returned to their standard home cages. We found that this model of brief, daily exposure to enrichment sufficiently increased both proliferation and differentiation in both control and arsenic-exposed animals regardless of perinatal exposure. The effect on control animals was as expected: significantly increased proliferation of 70%, likely due to exercise from use of the running wheel as suggested by work from van Praag, and significantly increased differentiation of 39%, likely due to exposure to novelty, other animals, and toys [59]. The use of both a running wheel and toys for enrichment has additive effects on adult neurogenesis and may likely account for the robust increase seen in both sets of animals [34]. While the increase in proliferation of arsenic-exposed animals after enrichment was not significant, we consider a 53% increase to be considerable. Qualitative evidence suggests that a substantial difference exists even if not statistically significant (Figure 4C). Two reasons for this lack of significance include 1) the inherent variability in measuring neurogenesis and 2) the fact that, while arsenic may influence NPC proliferation, this impact may only be observed as the lack of significant neurogenic response to the enrichment, as seen in other studies using teratogens (e.g., alcohol) [55]. Surprisingly, we found that brief exposure to enrichment led to increased differentiation (121%) in perinatal arsenic-exposed animals compared to their arsenic-exposed counterparts without exposure to enrichment. Cell counts show that arsenic-exposed animals have increased levels of differentiation after enrichment similar to those seen in control animals without exposure to enrichment. As expected, exposure to enrichment also increased mRNA levels of genes involved in proliferation and differentiation, including several growth and transcription factors (Table 3, 4; Figure 5). However, in control enriched animals versus control, no enrichment animals (Table 2), enrichment induces suppression of factors like Noggin, Cdkr1, Notch1, and Sox3, suggesting a shift from proliferation to differentiation in response to enrichment [34]. Exposure to enrichment significantly reversed aberrant gene expression in arsenic-exposed animals (Tables 3, 4; Figure 5), inducing upregulation of several genes important for axonogenesis, cell cycle dynamics, Notch signaling, and transcription factors. These results suggest that even though arsenic interferes with the number of differentiated cells in the dentate gyrus, the neurogenic morphological and genetic response to enrichment both is not impaired when using this particular playground model.

Evidence elucidating the importance of intact adult neurogenesis suggests that it is crucial for some types of hippocampal-dependent learning and memory, and ablation of adult neurogenesis results in learning and memory deficits similar to those observed in our arsenic model. We have demonstrated that exposure to 50 ppb arsenic, which is metabolized more efficiently in the mouse and models a 10 ppb arsenic exposure in humans, during all three trimesters of gestational and postnatal development induces hippocampal-dependent learning and memory deficits [8], [13]. Results presented here indicate it is likely that altered neurogenesis is one mechanism responsible for these deficits. This study is the first to show that developmental exposure to a low concentration of arsenic induces changes the number of differentiated adult neural progenitors in the subgranular zone of the hippocampus and aberrant expression of neurogenesis-related genes. Further studies are needed to elucidate mechanisms that produce these genetic and morphological changes we have observed.
